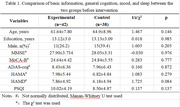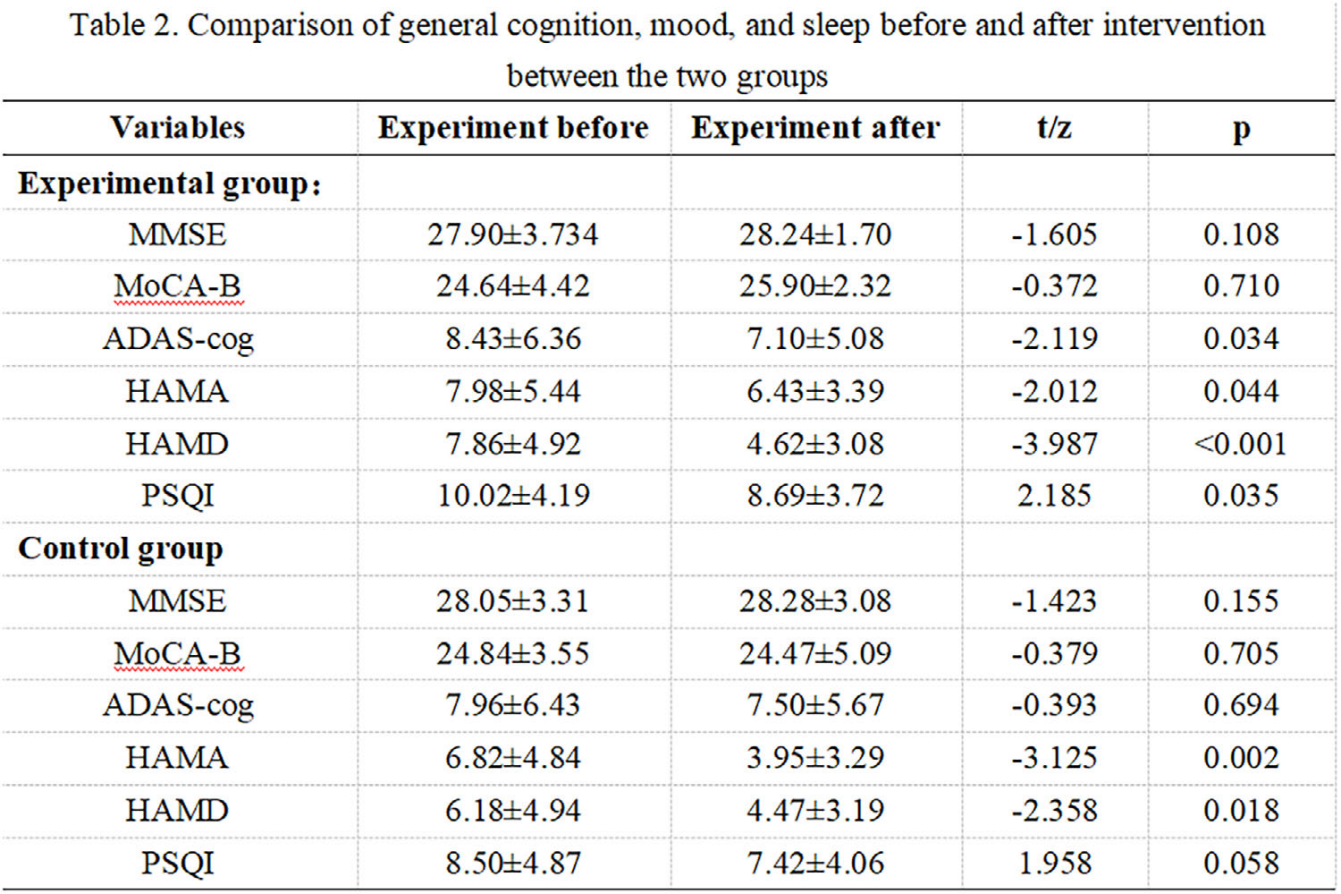# A study of the efficacy of 500nm blue‐green light therapy on cognition, mood, and sleep in prodromal Alzheimer's disease

**DOI:** 10.1002/alz.088968

**Published:** 2025-01-03

**Authors:** Qiansen Feng, Lei Chen, Linlin Li, Junlan Yang, Jiajing Wu, Yuchuan Yue, Ziqi Wang

**Affiliations:** ^1^ Nursing School of Zunyi Medical University, Zunyi China; ^2^ The Clinical Hospital of Chengdu Brain Science Institute, MOE Key Lab for Neuroinformation, School of Life Science and Technology, University of Electronic Science and Technology of China, Chengdu China; ^3^ The Fourth People’s Hospital of Pengzhou, Chengdu China

## Abstract

**Background:**

The study aimed to evaluate the effects of four weeks of 500 nm blue‐green light visual stimulation on cognition, mood, and sleep in patients with subjective cognitive decline (SCD) and mild cognitive impairment (MCI).

**Method:**

Eighty patients were recruited from the Memory Clinic. The experimental group comprised 42 cases (22 SCD and 20 MCI), while the control group comprised 38 cases (27 SCD and 11 MCI). The experimental group was treated with a 500nm blue‐green light (light spectrum intensity of 506 lux lm/m² and 230 µW/cm²) for 50 minutes in the morning for four weeks, while the control group received no treatment. General cognitive function was evaluated using the Mini‐mental State Examination (MMSE), Montreal Cognitive Assessment‐Basic (MoCA‐B), and Alzheimer’s Disease Assessment Scale‐cognitive subscale (ADAS‐cog), and mood was evaluated using the Hamilton Anxiety Scale (HAMA) and Hamilton Depression Scale (HAMD). The Pittsburgh Sleep Quotient Index (PSQI) was used to assess the sleep quality. Paired t‐test and Wilcoxon signed‐rank test were used to compare the differences in cognition, sleep, and mood between the experimental and the control group before and after the intervention.

**Result:**

There were no significant differences in baseline demographic information, cognition, mood, and sleep between the experimental and control groups (P>0.05). As shown in Table 1. After the intervention, the experimental group showed a significant reduction in the ADAS‐cog (P = 0.034), HAMA (P = 0.044), HAMD (P<0.001) and PSQI scores (P = 0.035). The control group showed a significant reduction in HAMA (P = 0.002) and HAMD (P = 0.018) scores. As shown in Table 2.

**Conclusion:**

Four weeks of 500 nm blue‐green light therapy significantly improved overall cognitive function and sleep quality in SCD and MCI patients.